# Are seminal vesicles a potential pitfall during pelvic exploration using point-of-care ultrasound (POCUS)?

**DOI:** 10.1186/s13089-021-00209-7

**Published:** 2021-03-01

**Authors:** Antoine Fasseaux, Philippe Pès, Françoise Steenebruggen, Florence Dupriez

**Affiliations:** 1 Department of Emergency Medicine, CHR Jolimont, 7100 La Louvière, Belgium; 2 Department of Emergency Medicine, CHU Nantes, Nantes University Hospital, 44000 Nantes, France; 3 Department of Emergency Medicine, Cliniques Universitaires Saint Luc, 1200 Brussels, Belgium

**Keywords:** Focused assessment with sonography for trauma (FAST), Pitfall, Emergency physician, Seminal vesicle, Point-of-care ultrasound, Ultrasound, Bedside ultrasound, Emergency ultrasound, Clinical ultrasound

## Abstract

**Background:**

Trauma is a major cause of death among the working population. Many countries have now adopted a structured approach to trauma management in which ultrasound is used as a primary evaluation tool. While its use has direct therapeutic benefits, many artifacts and pitfalls are inherent to the technique. Knowledge of the most frequently encountered pitfalls in practice could thus help reduce the risk of error and lead to more accurate trauma assessments.

**Objective:**

This study evaluates a potential pitfall caused by seminal vesicles during focused assessment with sonography for trauma examinations of the male pelvis performed by an emergency physician with experience in point-of-care ultrasound.

**Methods:**

We took five static and five dynamic (3-s loops) transverse ultrasound images of the pelvis in five healthy males. The images and videos were then incorporated into an online survey and emailed through the World Interactive Network Focused On Critical UltraSound (WINFOCUS) in France and the Ultrasound and Emergency Medicine (UEM) Organization in Belgium. A questionnaire asked anonymous participants to assess the presence of free fluid in the static and dynamic images and to share information about their training and experience in point-of-care ultrasound. To validate the static and dynamic images, the survey was sent to three external radiologists for independent verification.

**Results:**

A total of 191 individuals responded fully or partially to the survey, 114 of whom completed it. Among the 114 participants who completed the survey, the misinterpretation rate was 0.55 (95CI 0.51–0.60) for all static and dynamic ultrasound transverse pelvic views. The misinterpretation rate was 0.61 (95CI 0.55–0.66) and 0.50 (95CI 0.45–0.55) for static and dynamic ultrasound transverse pelvic views, respectively. The three external radiologists answered the questionnaire correctly without misinterpreting the survey ultrasound views.

**Conclusions:**

Seminal vesicles are a potential pitfall when interpreting transverse ultrasound images of the male pelvis in the context of point-of-care ultrasound.

## Background

Injuries are the leading cause of death for people aged between 1 and 44 years [1]. A structured approached to trauma management is followed in many countries using the Advanced Trauma Life Support (ATLS) protocol for the initial assessment of patients. The ATLS, Western Trauma Association, and Eastern Association for the Surgery of Trauma guidelines all support the use of ultrasound during the assessment of severe trauma [2–4]. As a result, clinical ultrasound use in emergency departments has increased considerably since its introduction more than 30 years ago [5]. Without interrupting management, bedside ultrasounds allow the rapid evaluation of patients to determine the presence of free fluid in the peritoneum or pericardium. Focused assessment with sonography for trauma (FAST) is a point-of-care ultrasound (POCUS) examination of four sites: Morrison’s pouch between the liver and right kidney, the splenorenal recess, the pelvic area, and the pericardial space [6].

Extended focused assessment with sonography for trauma (E-FAST) incorporates four additional areas: the two pulmonary apex and the two pulmonary bases. The exploration of these areas aims to identify the presence of free fluid in the pleura or clinically significant pneumothorax [7]. In unstable patients, E-FAST identifies the pericardial, peritoneal, or pleural location of the bleeding for faster surgical management [8]. FAST ultrasound examinations are now an integral part of emergency physician training [9]. Yet despite the large number of courses available around the world, ultrasound curricula suffer from a lack of standardization. Consequently, skill acquisition is less systematic, depending particularly on the types of cases encountered and the number of examinations carried out before clinical POCUS use [10].

Like other clinical ultrasound examinations, FAST can be prone to pitfalls and artifacts, leading to the misinterpretation of the acquired images. A recent meta-analysis involving 8635 patients showed a sensitivity of 0.74 (95CI 0.65–0.81) and a specificity of 0.96 (95CI 0.94–0.98) when E-FAST is performed by an emergency physician [11].

In some ultrasound curricula, seminal vesicles are reported as a source of misinterpretation when assessing the male pelvis [12]. Located behind the bladder, seminal vesicles have a hypoechoic appearance that can be mistaken for free fluid. Misinterpretation of a FAST could have a direct therapeutic impact. A literature search did not reveal any evidence of seminal vesicles as a source of pitfall during FAST. The main goal of this study is therefore to verify whether seminal vesicles may be a source of false-positive POCUS results performed by an emergency physician trained in POCUS.

## Methods

Five healthy male volunteers were scanned using a Philips Sparq® and a C6 2–6 MHz convex probe. The abdomen preset of the ultrasound machine was chosen for the ultrasound examination and image registration. Each volunteer was scanned to obtain static and dynamic ultrasound transverse views of the pelvis. The dynamic image was a 3-s loop showing transverse ultrasound images of the pelvis from the pubic symphysis toward the apex of the bladder, obtained by fanning the probe. The seminal vesicles were visible on each ultrasound image. The static and dynamic images were incorporated into an online survey via SurveyMonkey® using a high image resolution in JPEG format. After validation, the survey was sent by email to the networks of the World Interactive Network Focused On Critical UltraSound (WINFOCUS) in France and the Ultrasound and Emergency Medicine (UEM) Organization in Belgium. Only the anonymous participants who described themselves as familiar with FAST ultrasound were given the opportunity to complete the survey in French or in Dutch. (available at https://fr.surveymonkey.com/r/MQ297W2) After recording the demographic and professional (Table 1) profile and clinical ultrasound background of the participating emergency physicians, they were asked whether they saw free fluid on the recorded static and dynamic images. For the analysis of the ultrasound images, apart from the gender of the volunteers and the ultrasound view setting (i.e., male transverse pelvic view), no other clinical information was given to participants. Only the results of fully completed questionnaires were included in the study. The participants were not given the opportunity to repeat the survey or review the images and change their responses after seeing the next question. To independently validate the selected static and dynamic ultrasound images, the survey was taken by three external radiologists.

**Table 1:** Profile of participants

Profile	Total (*n* = 114)
Experience in emergency medicine	
< 5 years	38 (33%)
5–10 years	34 (29.83%)
10–20 years	30 (26.32%)
20–30 years	10 (8.77%)
> 30 years	2 (1.75%)
Total	114 (100%)
Ultrasound training	
None	1 (0.88%)
Short course	61 (53.51%)
Validated short course	6 (5.26%)
Long course	29 (25.44%)
Multiple training courses (2 or more)	17 (14.91%)
Total	114 (100%)
Ultrasound frequency of use	
Daily	71 (62.28%)
Weekly	37 (32.46%)
Monthly	4 (3.51%)
Rarely	2 (1.75%)
Total	114 (100%)
Ultrasound availability at work	
Yes	111 (97.37%)
No	3 (2.63%)
Total	114 (100%)

For the statistical analysis, the calculations were performed using the software Excel® (Microsoft, Redmond (Washington), United States). A paired data t-test was used to compare the results of the static and dynamic sections.

## Results

Between February 1 and March 31, 2020, 191 people responded to the survey. Overall, 77 people were excluded from the study because they did not complete the questionnaire, while 114 completed it entirely. Table 1 reports the profile of participants.

The misinterpretation rate was 0,55 (95CI 0.51–0.60) for all static and dynamic ultrasound transverse pelvic views. The misinterpretation rate was 0.61 (95CI 0.55–0.66) and 0.50 (95CI 0.45–0.55) for static and dynamic ultrasound transverse pelvic views, respectively. The difference in misinterpretation rate for static and dynamic images was 0.53 (*p*-value 0.0001).

Given the large heterogeneity in the number of participants in the different subgroups, no statistically significant correlation was found in terms of professional experience, frequency of ultrasound use, or training background. The misinterpretation rates according to the different subgroups are described in Table 2. The three external radiologists all correctly answered the questionnaire and did not mistake the seminal vesicles for free fluid.

**Table 2:** Mean misinterpretation rate in participant subgroups

	Total (*n* = 10)	Static views (*n* = 5)	Dynamic views (*n* = 5)
All categories combined	5.54 ± 0.46	3.04 ± 0.28	2.51 ± 0.24
Experience in emergency medicine			
< 5 years	5.55 ± 0.78	3.06 ± 0.48	2.49 ± 0.42
5–10 years	5.58 ± 0.83	3.06 ± 0.51	2.52 ± 0.45
10–20 years	5.58 ± 0.90	3.06 ± 0.55	2.53 ± 0.49
20–30 years	5.59 ± 1.75	3.05 ± 1.08	2.50 ± 0.93
> 30 years	5.86 ± 17.44	3.14 ± 12.41	2.72 ± 9.57
Ultrasound training			
None (1 response)	9	5	4
Short course	5.56 ± 0.62	3.05 ± 0.38	2.51 ± 0.33
Validated short course	5.65 ± 2.56	3.06 ± 1.57	2.59 ± 1.33
Long course	5.65 ± 0.91	3.10 ± 0.56	2.55 ± 0.49
Multiple training courses (2 or more)	5.18 ± 1.35	2.88 ± 0.80	2.29 ± 0.65
Ultrasound frequency of use			
Daily	5.59 ± 0.56	3.07 ± 0.34	2.51 ± 0.31
Weekly	5.54 ± 0.81	3.04 ± 0.50	2.50 ± 0.43
Monthly	5.71 ± 3.79	3.05 ± 2.31	2.66 ± 2.04
Rarely	6.00 ± 21.18	3.30 ± 10.42	2.70 ± 12.02
Ultrasound available at work			
Yes	5.54 ± 0.46	3.04 ± 0.28	2.51 ± 0.25
No	5.72 ± 5.78	3.12 ± 3.55	2.61 ± 3.17

## Discussion

Many emergency departments use ultrasound on a daily basis to assess patients. The FAST protocol and its variant e-FAST allow the rapid assessment of severe trauma patients. However, there is a lack of standardization in the different training programs and especially in the teaching of the FAST protocol [10].

**Fig. 1:**
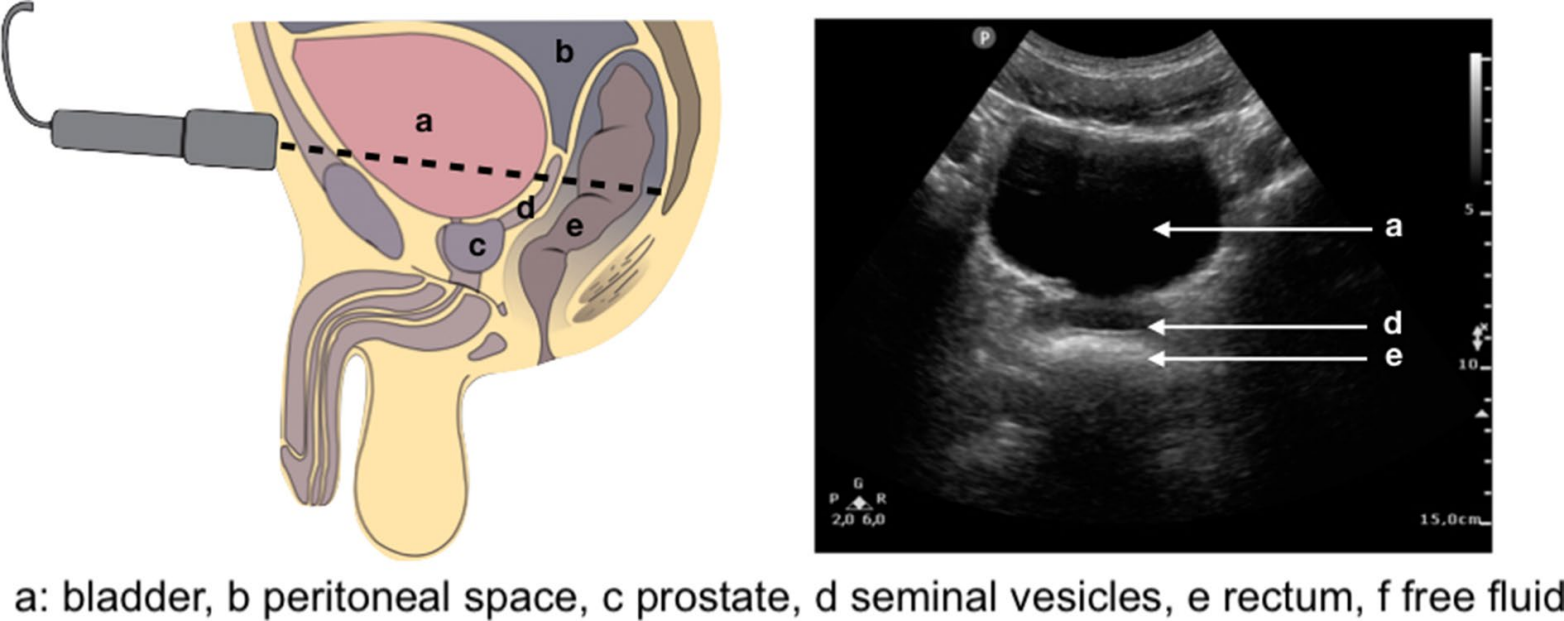
Transverse ultrasound view of the male pelvis—probe oriented towards the seminal vesicles

**Fig. 2:**
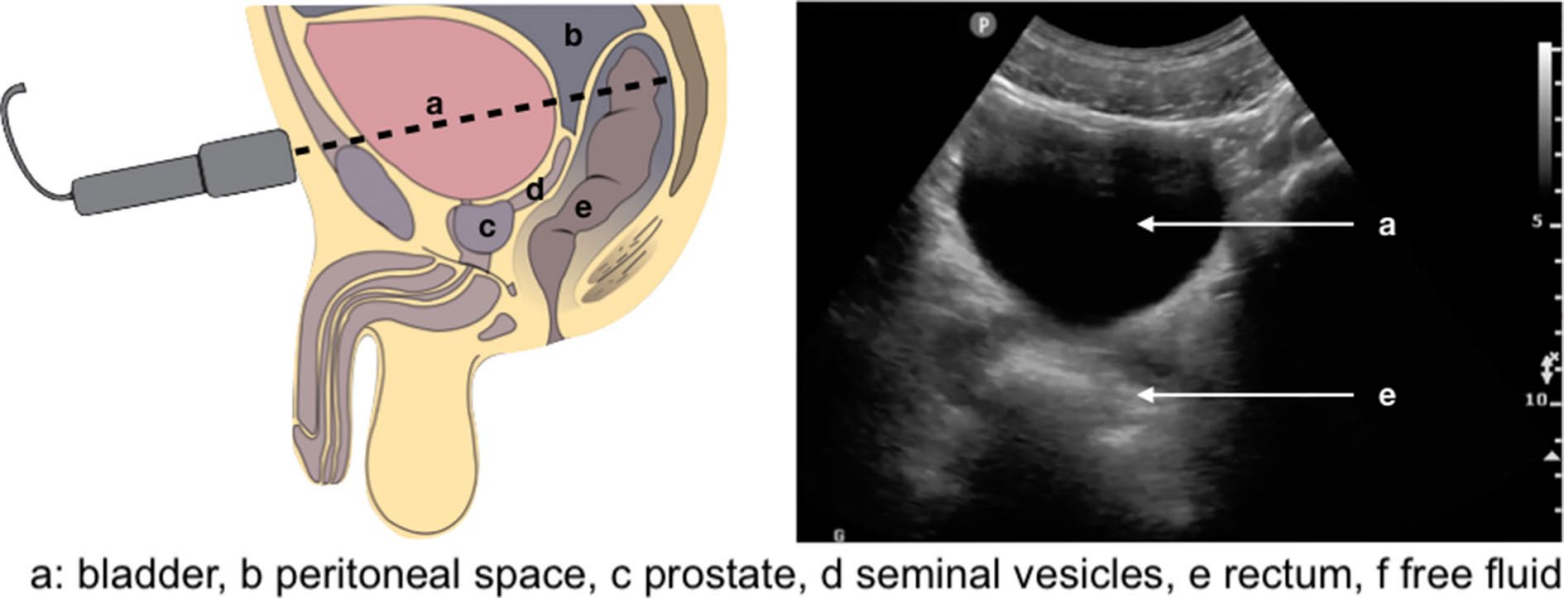
Transverse ultrasound view of the male pelvis—probe oriented towards the peritoneal space

**Fig. 3:**
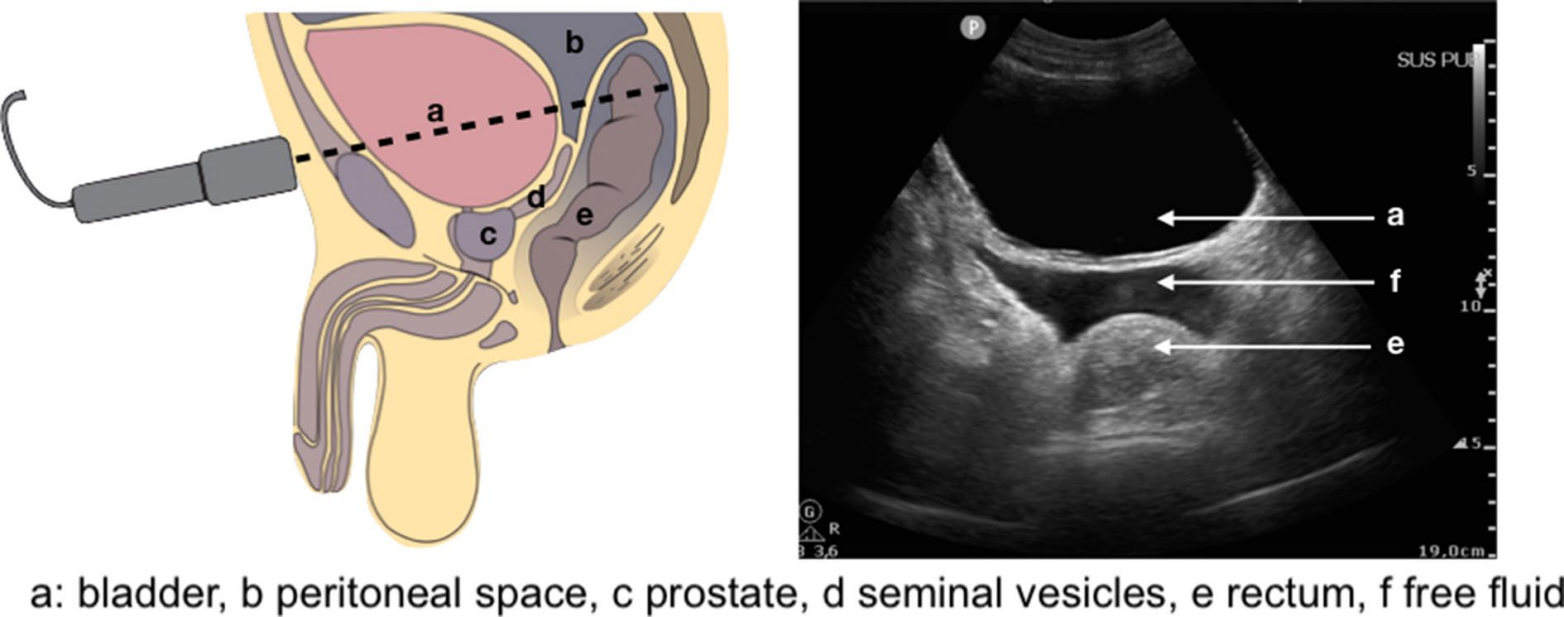
Transverse ultrasound view of the male pelvis with free fluid—probe oriented towards the peritoneal space

Located in the retroperitoneum, the seminal vesicles contain fluid that can have a hypoechoic appearance in the ultrasound images of the male pelvis (Fig. 1, Additional file 1: video S1). Although poorly studied, this hypoechoic structure may be misinterpreted as free fluid. In male patients, free fluid is located in the Douglas pouch, never next to or directly above the prostate, but rather more cranially at the level of the bladder. Figure 2 shows a transverse ultrasound view of the male pelvis above the seminal vesicles, whereas Fig. 3 and Additional file 2: video S2 show a pathological ultrasound view of the male pelvis with free fluid. Our study suggests that the seminal vesicles are a potential POCUS pitfall when assessing the presence of free fluid in the male pelvis. Among the ultrasound images included in the survey, only the seminal vesicles could be the source of misinterpretation for free fluid. The completion of the survey by three external radiologists to confirm the quality of the chosen ultrasound transverse pelvic views gives methodological strength to this study.

Although participants were given information about the view and location of the ultrasound images as well as the gender of the volunteers, seminal vesicles were not specially mentioned in order to avoid influencing responses. All included participants described themselves as familiar with the FAST protocol, and only one participant had not received clinical ultrasound training. Participants’ knowledge may thus be considered representative of ultrasound training courses, as they had a heterogeneous background in ultrasound imaging. This study also significantly demonstrates that dynamic examinations of the pelvic region reduce the number of misinterpretations compared to static images. This enlightens the importance of using dynamic, rather than static, assessment of ultrasound images during a FAST examination. This dynamic assessment should cover the region. As the participants were not given the opportunity to repeat the survey or return to a previous question, their initial responses could not be changed after the subsequent viewing of static or dynamic images. Since this study only aims to highlight the false-positive rate, no pathological image was included in the survey.

The absence of a pathological image may nevertheless have influenced the certainty index of participants’ responses, as they may have interpreted images according to the following yes–no question: is free fluid visible in this transverse ultrasound image of the male pelvis? However, the survey used carefully chosen sentences to avoid influencing participants about the possible absence or presence of free fluid.

The exclusive use of a transverse section view without a longitudinal view may be cited as a limitation of this study. Indeed, the e-FAST protocol strictly recommends the use of a transverse view along with a longitudinal view to assess the presence of free fluid in the pelvis [13]. Complementary studies should therefore clarify whether the additional use of a longitudinal ultrasound view decreases the number of misinterpretations.

Another limitation of this study relates to its setting. Indeed, a questionnaire is far removed from the clinical setting. However, responses provided without the stress of a pathological setting could be considered even more accurate than answers given in the stress of a severe trauma assessment. Complementary studies are nevertheless required to avoid this bias. FAST and, more broadly, POCUS aim to clinically interpret the images obtained as part of a comprehensive approach to patient care. In addition, ultrasound is an operator-dependent examination, which gives the questionnaire setting of this study another substantial bias. This bias can be partially attenuated given that the quality of the images was independently and successfully validated by three radiologists.

Another limitation of this work is the selection bias due to the inclusion of physicians registered in the database of two ultrasound training providers in France and Belgium. This does not account for the fact that participants received training in these organizations. Indeed, the mailing lists of the organizations included participants with other courses’ background, although their proportion could not be assessed. Both organizations only offer short ultrasound training programs. The background heterogeneity is therefore substantial in the different subgroups.

Given the COVID-19 crisis, the follow-up email that was initially planned could not be returned by these two organizations.

## Conclusion

The FAST ultrasound protocol is now standard in the management of trauma patients in many countries. Seminal vesicles can nevertheless be a source of misinterpretation when evaluating transverse ultrasound images of the male pelvis. However, this is significantly diminished by the interpretation of dynamic views. This potential pitfall should be addressed in the various teaching methods and available curricula. Ultrasound instructors should also stress the importance of the dynamic assessment of the pelvis using a fanning movement. Further studies are required to determine whether the addition of a longitudinal view helps decrease the incidence of this newly studied pitfall. We believe that ultrasound curricula available around the world should introduce seminal vesicles as a potential pitfall when using POCUS.

## Supplementary Information

**Additional file 1: Video S1.** Normal transverse ultrasound view of the male pelvis.

**Additional file 2: Video S2.** Pathological transverse ultrasound view of the male pelvis—free fluid.

## Data Availability

The datasets used and/or analyzed during the current study are available from the corresponding author on reasonable request.

## Abbreviations

POCUS: Point-of-care ultrasound
WINFOCUS: World Interactive Network Focused On Critical UltraSound (WINFOCUS) UEM: Ultrasound and Emergency Medicine
FAST: Focused assessment with sonography for trauma
ATLS: Advanced Trauma Life Support
E-FAST: Extended focused assessment with sonography for trauma
COVID-19: Coronavirus disease 2019
